# Reduction of Onset Delay in Functional Near-Infrared Spectroscopy: Prediction of HbO/HbR Signals

**DOI:** 10.3389/fnbot.2020.00010

**Published:** 2020-02-18

**Authors:** Amad Zafar, Keum-Shik Hong

**Affiliations:** ^1^School of Mechanical Engineering, Pusan National University, Busan, South Korea; ^2^Department of Electrical Engineering, University of Wah, Wah Cantonment, Pakistan; ^3^Department of Cogno-Mechatronics Engineering, Pusan National University, Busan, South Korea

**Keywords:** hemodynamic response, prediction, tracking, vector phase analysis, brain-machine interface (BMI), functional near-infrared spectroscopy (fNIRS)

## Abstract

An intrinsic problem when using hemodynamic responses for the brain-machine interface is the slow nature of the physiological process. In this paper, a novel method that estimates the oxyhemoglobin changes caused by neuronal activations is proposed and validated. In monitoring the time responses of blood-oxygen-level-dependent signals with functional near-infrared spectroscopy (fNIRS), the early trajectories of both oxy- and deoxy-hemoglobins in their phase space are scrutinized. Furthermore, to reduce the detection time, a prediction method based upon a kernel-based recursive least squares (KRLS) algorithm is implemented. In validating the proposed approach, the fNIRS signals of finger tapping tasks measured from the left motor cortex are examined. The results show that the KRLS algorithm using the Gaussian kernel yields the best fitting for both ΔHbO (i.e., 87.5%) and ΔHbR (i.e., 85.2%) at *q* = 15 steps ahead (i.e., 1.63 s ahead at a sampling frequency of 9.19 Hz). This concludes that a neuronal activation can be concluded in about 0.1 s with fNIRS using prediction, which enables an almost real-time practice if combined with EEG.

## Introduction

Similar to functional magnetic resonance imaging and electroencephalography (EEG), functional near-infrared spectroscopy (fNIRS) is a non-invasive neuroimaging technique that measures hemoglobin oxygenation changes in the brain (Kato et al., 1993; Villringer et al., 1993). fNIRS can measure the absolute as well as relative concentration changes of oxyhemoglobin (HbO/ΔHbO) and deoxyhemoglobin (HbR/ΔHbR) using multiple near-infrared lights within the range of 650~1,000 nm (Pellicer and Del Carmen Bravo, 2011; Boas et al., 2014; Nguyen et al., 2016). It offers several advantages, including acceptable temporal and spatial resolution (Hong and Naseer, 2016; Nguyen and Hong, 2016), portability, and low cost (Ferrari and Quaresima, 2012). With these advantages, fNIRS has successfully demonstrated its potential as a viable neuroimaging tool for applications to the health care industry (Hong and Yaqub, 2019), neurological disorders (Obrig, 2014; Ghafoor et al., 2019; Yang et al., 2019), psychiatric disorders (Ohi et al., 2017), behavioral and cognitive development (Watanabe et al., 2017; Yaqub et al., 2018), and brain-computer interfaces (BCIs) (Nicolas-Alonso and Gomez-Gil, 2012; Naseer and Hong, 2015; Schudlo and Chau, 2018; Shin and Im, 2018).

The measured fNIRS signals (i.e., ΔHbO, ΔHbR) can be categorized into three durations (Frostig et al., 1990; Ernst and Hennig, 1994): (i) the initial dip, which represents the early extraction of oxygen by the nearby active neurons causing the ΔHbO/ΔHbR to decrease/increase, (ii) the conventional hemodynamic response (HR) that is the large increase in cerebral blood flow (CBF) resulting in an increase/decrease in ΔHbO/ΔHbR, respectively, and (iii) the undershoot before going back to the rest state. The changes in ΔHbO/ΔHbR upon the functional stimulation can be translated into meaningful commands for BCI applications (Matthews et al., 2008). These converted signals can be further used to actuate external devices such as robotic arm/leg or wheelchairs for improving the quality of patient lives (Mcfarland and Wolpaw, 2010, 2011; Ortiz-Rosario and Adeli, 2013; Yazdani et al., 2018). In particular, fNIRS devices are portable and have shown great potential for BCI applications. The main limitation of fNIRS for BCI is its slow nature of the HR and the inherent delay from the onset of the neuronal activity (Jasdzewski et al., 2003; Cui et al., 2010; Ahn and Jun, 2017), which restricts its use for online BCI applications as well as hybridization with other rapid techniques such as EEG (Jiao et al., 2018; Li et al., 2018; Yang et al., 2018), magnetoencephalography, etc. Because of this limitation, various features in different temporal windows of 0–5, 2–7, 0–10, 0–15, 0–17, and 0–20 s were used in multi-class classification algorithms to classify HRs associated with the same or different brain regions for fNIRS-BCI applications (Power et al., 2011; Khan et al., 2014; Schudlo and Chau, 2014; Gateau et al., 2015; Khan and Hong, 2015; Hong et al., 2017; Shin et al., 2017; Liu et al., 2018; Yi et al., 2018). Thus far, the features frequently used from these windows include signal mean, signal slope, signal peak, skewness, kurtosis, variance, standard deviation, number and sum of peaks, root mean square, median, etc. (Hwang et al., 2016; Naseer et al., 2016; Liu and Hong, 2017; Hong et al., 2018b; Wibowo et al., 2018).

Another means of addressing this delay is to utilize the initial dip for fast fNIRS-BCI applications. The initial dip is an early change in oxygenation prior to any subsequent increase in CBF, which is spatially more specific to the site of neuronal activity (Vanzetta and Grinvald, 2008; Hong and Zafar, 2018). However, there is also a time lag in detecting the initial dip (Hong and Naseer, 2016). A previous study by Hong and Naseer (2016) showed that the initial dip could be detected using a vector phase diagram with a single threshold circle. The vector phase diagram is a computationally efficient method to detect both the initial dip and the HR by displaying the trajectories of ΔHbO and ΔHbR, as orthogonal components, in the ΔHbO-ΔHbR polar coordinates (Oka et al., 2015). It was further proposed to use *q*-step-ahead prediction scheme in combination with the vector phase diagram to reduce the time lag in detecting the initial dip. They showed that the initial dip could be detected in 0.9 s using the *q*-step-ahead prediction scheme, showing high potential for BCI applications. Later, Zafar and Hong (2017) attempted to find the features and temporal window size for classifying the initial dip duration in fNIRS signals of different mental tasks. They showed that the running temporal window size for fNIRS could be reduced from 5 to 2.5 s using initial dip features (i.e., signal mean and signal minimum) in the classification process. Li et al. (2017) also used the mean value of ΔHbO and ΔHbR signals in the 0–2 s window as an initial dip feature and achieved 85.5% classification accuracy for the classification of left- and right-hand movements. Similarly, Khan and Hong (2017) used signal minimum as an initial dip feature and achieved a classification accuracy of 75.6% in classifying four mental tasks in a reduced window size (i.e., 0–2 s).

**Figure 1:**
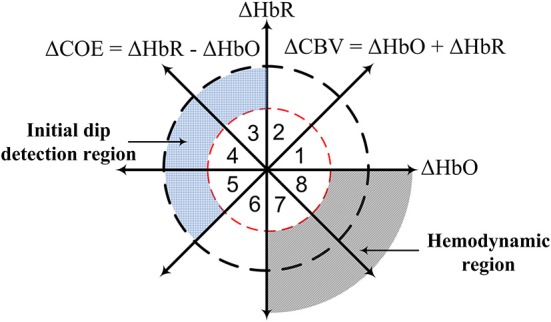
Concept of vector phase diagram with dual threshold circles (Hong and Zafar, 2018; Zafar and Hong, 2018).

The use of dual threshold circles in the vector phase diagram was proposed to improve the detection of both initial dip and the conventional HR (Zafar and Hong, 2018), see Figure 1. The threshold circles in the vector phase analysis helps to minimize the false detection of resting-state fluctuation and large fluctuations of ΔHbO and ΔHbR signals during the task period. The radius of the inner circle was set to the maximum HbO during the resting state, and the radius of the outer circle was set to the sum of the radius of the inner circle and 30% of the peak value of the main HR. The peak value of the HR was determined through the averaging over trials measured in the training phase. They showed that the use of dual threshold circles in the vector phase diagram resulted in an enhancement of the classification accuracies of two-finger tapping tasks. They also used the signal mean and the minimum signal value in 0–2.5 s time window to classify two-finger tapping tasks. However, windows of 0–2 s or 0–2.5 s are still too large for real-time BCI applications and hybridization of fNIRS with other rapid techniques such as EEG. Furthermore, the previously mentioned *q*-step-ahead prediction scheme by Hong and Naseer (2016) to reduce the delay was an offline analysis, and the validity of the predicted signals with multiple steps was not examined. Knowing the maximal prediction size of the *q*-step-ahead prediction method is important because the error of the predicted signals increases significantly with the increase of the number of step sizes. In addition, for real-time BCI applications, an online scheme is required to reduce the onset delay in fNIRS signals.

In this study, the use of a kernel recursive least squares algorithm (KRLS) is proposed for the *q*-step-ahead prediction of fNIRS signals. Three most commonly used kernels (i.e., Gaussian, polynomial, and sigmoid) are tested to compare the errors in the predicted fNIRS signals. Then, the effectiveness of the proposed prediction scheme was evaluated using fNIRS signals of finger tapping tasks measured from the left motor cortex of eleven subjects. The results of the proposed scheme were compared with those of the commonly used recursive least squares (RLS) algorithm. This paper further presents the applicability of the *q*-step-ahead prediction scheme to reduce the time lag in detecting the initial dips in fNIRS signals.

## Methods

### Brain Activity Model and Kernel Recursive Least Square

**Figure 2:**
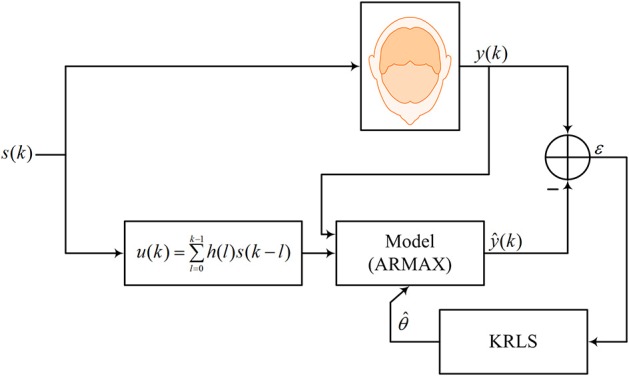
Online estimation/prediction scheme.

In this paper, a brain activity is modeled in a linear form using the autoregressive moving average with exogenous signals (ARMAX) model as follows.

(1) $$\begin{array}{l} y^{i}(k)\;=\sum_{n\;=\;1}^{n_{o}}a_{n}^{i}y^{i}(k-n)+\sum_{m=1}^{m_{o}}b_{m}^{i}(m)u(k-m)\\ \;+\sum_{p=1}^{p_{o}}c_{p}^{i}(p)w^{i}(k-p)+c_{o}\cdot 1+\varepsilon^{i}(k) \end{array}$$

where *i* represents the channel number; *y* is the measured ΔHbO/ΔHbR; *u* is the desired hemodynamic response function (dHRF); *w* is the physiological noise; ε is the zero-mean Gaussian noise; *a*_n_, *b*_m_, *c*_p_, and *c*_o_ are unknown coefficients that are recursively estimated; and *n*_o_, *m*_o_, and *p*_o_ are the orders of the system, input, and exogenous signals, respectively. For fNIRS, the exogenous signal *w* consists of specifically three sinusoidal signals representing cardiac, Mayer, and respiration related physiological noises (Abdelnour and Huppert, 2009; Nguyen H.-D. et al., 2018). Also, the exogenous signals can be dropped out in the estimation process (i.e., *p*_o_ = 0) if the prediction/tracking of the measured signal is focused. Nevertheless, the fNIRS signals were low- and high-pass filtered to minimize the effect of the physiological noises before the estimation process. Equation (1) can be written in a simplified vector form as follows.

(2) $$\begin{array}{l} y^{i}\left(k\right)=\varphi^{T}\left(k\right)\theta^{i}\left(k\right)+e^{i}\left(k\right) \end{array}$$

(3) $$\begin{array}{l} \varphi^{T}(k)=[y(k-1)\cdots y(k-n_{o})\\ \;u(k-1)\cdots u(k-m_{o})\;w(k-1)\cdots w(k-p_{o})\;1] \end{array}$$

(4) $$\theta^{i}(k)=\left[\begin{array}{llll} a_{1}^{i}\cdots a_{n_{o}}^{i} & b_{1}^{i}\cdots b_{m_{o}}^{i} & c_{1}^{i}\cdots c_{p_{o}}^{1} & c_{o} \end{array}\right]$$

where φ(*k*) ∈ ℜ^(*n* + *m* + *p* + 1) × 1^ is the regression vector and superscript *T* stands for the transpose operator. Figure 2 shows the estimation/prediction scheme.

In this study, dHRF [i.e., *u*(*k*)] was generated by convolving the canonical HRF (cHRF), denoted by *h*(*k*), with a stimulation period, *s*(*k*), as follows.

(5) $$\begin{array}{l} u\left(k\right)=\overset{k-1}{\underset{l=0}{\sum }}h\left(l\right)s\left(k-l\right), \end{array}$$

(6) $$s\left(k\right)=\{\begin{array}{c} 1,\text{ if }k\in task,\\ 0,\text{ if }k\in rest, \end{array}$$

where *task* and *rest* represent the task period and the rest period, respectively (task = 10 s and rest = 20 s in this study). cHRF was generated as a linear combination of three gamma functions by the following equation (Shan et al., 2014).

(7) $$\begin{array}{l} h\left(k\right)=\overset{3}{\underset{j=1}{\sum }}A_{j}\frac{k^{\alpha_{j}-1}\beta_{j}^{\alpha_{j}}e^{-\beta_{j}k}}{\Gamma \left(\alpha_{j}\right)}, \end{array}$$

where *j* represents the number of gamma functions, *A*_*j*_ is the amplitude, α_*j*_ and β_*j*_ tune the shape and the scale, respectively, and *k* is the time step (in this work, *A*_1_ = −1.5, *A*_2_ = 7, *A*_3_ = −2, α_1_ = 1.5, α_2_ = 6, α_3_ = 16, and β_1_ = β_2_ = β_3_ = 1 were used). The unknown coefficients in Equation (2) are estimated and updated using the KRLS based on the optimization of the cost function given by

(8) $$\begin{array}{l} \underset{\theta \left(k\right)}{\min}J_{KRLS}=\overset{N}{\underset{k=1}{\sum }}\lambda^{N-k}{\left|y\left(k\right)-\kappa {\left(\varphi \left(k\right),\;\cdot \right)}^{T}\theta \left(k-1\right)\right|}^{2}\\ \;+R\lambda^{N}\|\theta \left(k-1\right){\|}_{H}^{2}, \end{array}$$

(9) $$\begin{array}{l} \Phi (k)=[\kappa (\varphi (1),\;\cdot )\;\kappa (\varphi (2),\;\cdot )\\ \;\cdots \;\kappa (\varphi (k),\;\cdot ){]}^{T} \end{array}$$

where κ represents the Mercer kernel, Φ is the kernel matrix of all *k* input data points, *R* is a positive number known as the regularization parameter, *H* represents the reproducing kernel Hilbert space (RKHS) associated with the Mercer kernel, and λ (0.98 in this study) is the forgetting factor. The performances of the following three most commonly used kernels in improving the prediction of the fNIRS signals are tested (Muller et al., 2001):

(i) Gaussian kernel

(10) $$\begin{array}{l} \kappa \left(\varphi ,\varphi^{\prime }\right)=\exp\left(-\frac{{\left||\varphi -\varphi^{\prime }|\right|}^{2}}{2\sigma^{2}}\right) \end{array}$$

where σ is a scaling factor, and φ′represents the new upcoming data.

(ii) Polynomial kernel

(11) $$\begin{array}{l} \kappa \left(\varphi ,\varphi^{\prime }\right)={\left(\varphi^{T}\varphi^{\prime }+c\right)}^{p} \end{array}$$

where *c* is a non-negative constant, and *p* is the order of the polynomial kernel.

(iii) Sigmoid kernel

(12) $$\begin{array}{l} \kappa \left(\varphi ,\varphi^{\prime }\right)=\tanh\left(s\left(\varphi^{T}\varphi^{\prime }\right)+t\right) \end{array}$$

where *s* and *t* are suitable non-negative constants.

The basic idea is to map input data points to a high dimensional feature space (i.e., RKHS). This process allows the transformation of linear inner products into RKHS by simply changing their inner product into kernels (Schölkopf and Smola, 2002; Liu et al., 2010). The transformed feature space is then solved using the linear algorithm. The advantage of kernel-based algorithms is that they have a unique global solution that can be derived by solving a convex optimization problem (Chen et al., 2014). Furthermore, if data show a non-linear relationship, linear regression techniques cannot model them adequately. The kernel method can address this issue by moving to another feature space that is more likely to correspond to a linear model. However, the kernel method suffers from the overfitting problem because the Hilbert space induces high dimensionality of data. To address the issue of overfitting, the solution is penalized by limiting it to the L2 norm, as shown in Equation (8) (Evgeniou et al., 2000; Pillonetto et al., 2014), which is solved and updated as follows (Liu et al., 2010).

(13) $$\begin{array}{l} \theta \left(k\right)=\Phi \left(k\right){\left[R\lambda +\Phi {\left(k\right)}^{T}\Phi \left(k\right)\right]}^{-1}y\left(k\right),\\ \theta \left(k\right)=\Phi \left(k\right)a\left(k\right),\;a\left(k\right)=Q\left(k\right)y\left(k\right), \end{array}$$

(14) $$\begin{array}{l} Q\left(1\right)={\left[R\lambda +\kappa \left(\varphi \left(1\right),\varphi \left(1\right)\right)\right]}^{-1},\;a\left(1\right)=Q\left(1\right)y\left(1\right), \end{array}$$

(15) $$\begin{array}{l} K(k)=K{\left(k-1\right)}^{T}\kappa (\varphi (k),\;\cdot )=\left[\kappa (\varphi (1),\;\varphi \left(k\right)\right),\;\cdots ,\\ \;{\kappa \left(\varphi (k-1),\varphi \left(k\right)\right)]}^{T}, \end{array}$$

(16) $$\begin{array}{l} z\left(k\right)=Q\left(k-1\right)K\left(k\right), \end{array}$$

(17) $$\begin{array}{l} \delta \left(k\right)=R\lambda +\kappa \left(\varphi \left(k\right),\varphi \left(k\right)\right)-z^{T}\left(k\right)K\left(k\right), \end{array}$$

(18) $$\begin{array}{l} Q\left(k\right)=\delta^{-1}\left(k\right)\left[\begin{array}{cc} Q\left(k-1\right)\delta \left(k\right)+z\left(k\right)z^{T}\left(k\right) & -z\left(k\right)\\ -z\left(k\right) & 1 \end{array}\right], \end{array}$$

(19) $$\begin{array}{l} e\left(k\right)=y\left(k\right)-K^{T}\left(k\right)a\left(k-1\right), \end{array}$$

(20) $$\begin{array}{l} a\left(k\right)=\left[\begin{array}{c} a\left(k-1\right)-z\left(k\right)\delta^{-1}\left(k\right)e\left(k\right)\\ \delta^{-1}\left(k\right)e\left(k\right) \end{array}\right]. \end{array}$$

As the kernel matrix grows linearly with the number of observations, the computational complexity of KRLS increases. The complexity is reduced by using the approximate linear dependency (ALD) criterion (Engel et al., 2004). The KRLS-ALD algorithm has been implemented using the Matlab^TM^ kernel adaptive filtering toolbox (Van Vaerenbergh and Santamaría, 2013; Van Vaerenbergh, 2017).

Accordingly, using Equation (2), the estimated brain activity model can be represented as

(21) $$\begin{array}{l} {\hat{y}}^{i}\left(k\right)=\varphi^{T}\left(k\right){\hat{\theta }}^{i}\left(k\right)+e^{i}\left(k\right). \end{array}$$

For *q*-step-ahead prediction, Equation (21) can be written as follows.

(22) $$\begin{array}{l} {\hat{y}}^{i}\left(k+q\right)=\varphi^{T}\left(k+q\right){\hat{\theta }}^{i}\left(k\right)+e^{i}\left(k\right). \end{array}$$

The performance of the algorithm was tested using the percentage fitting (%FIT) criterion as follows (Pillonetto et al., 2014).

(23) $$\begin{array}{l} \text{FIT}=100\left(1-\sqrt{\frac{\overset{N}{\underset{k=1}{\sum }}{\left(y^{i}\left(k\right)-{\hat{y}}^{i}\left(k\right)\right)}^{2}}{\overset{N}{\underset{k=1}{\sum }}{\left(y^{i}\left(k\right)-mean\left(y^{i}\right)\right)}^{2}}}\right). \end{array}$$

The performance criterion in (23) quantifies how much of the variance of *y* is captured by the *q*-step-ahead predicted signal (Pillonetto et al., 2014). Furthermore, %FIT criteria measure how accurately the *q*-step-ahead predicted signals are estimated.

### Experimental Data

**Figure 3:**
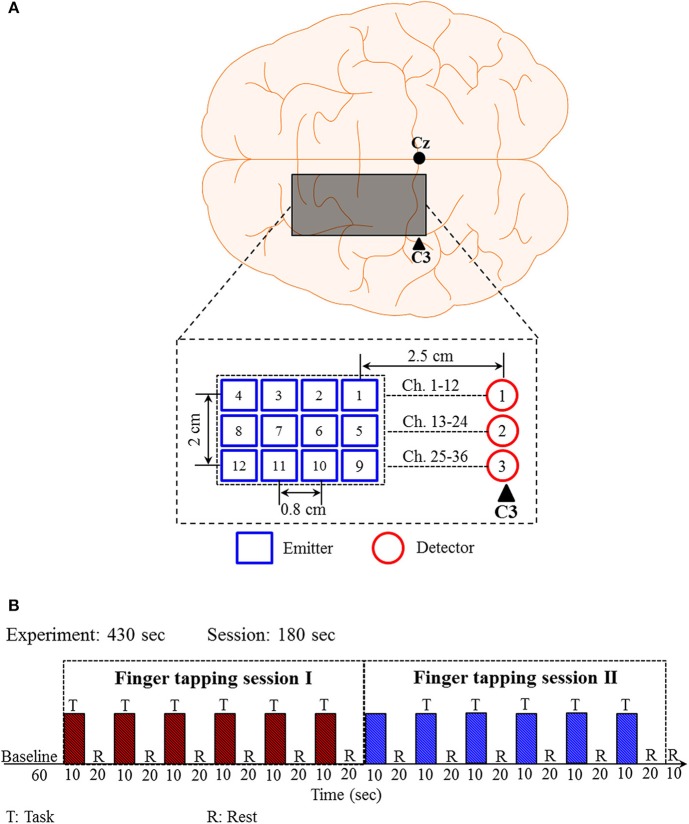
Emitter-detector placement and experimental paradigm for the right-hand finger tapping task: **(A)** Emitter-detector placement and their distances, **(B)** experimental paradigm.

Previously published experimental data (Zafar and Hong, 2018) of right-hand (thumb and little) finger tapping sessions from 11 subjects were used for validating the proposed *q*-step-ahead prediction scheme. Brain signals generated by the finger-tapping were acquired from the left motor cortex using the frequency domain fNIRS system (ISS Imagent, ISS Inc.) at a sampling rate of 9.19 Hz. The electrode placement and the corresponding emitter-detector distances are shown in Figure 3A. A total of 36 channels were formed using emitter-detector pairs.

The experimental paradigm is shown in Figure 3B. The experimental paradigm consists of two sessions of finger tapping tasks. A session is composed of six trials of 30 s. Each trial includes a 10 s activity task followed by a 20 s rest. During the task period, the subjects were instructed to tap their right-hand finger as fast as they could without paying attention to the number of taps. The raw data (ΔHbO and ΔHbR) obtained from the ISS Imagent data acquisition and analysis software (ISS-Boxy) were pre-processed to remove physiological noises related to respiration, cardiac, and low-frequency drift signals. Fourth-order Butterworth low- and high-pass filters with cutoff frequencies of 0.15 and 0.01 Hz, respectively, were used to minimize the respiration, cardiac, and low-frequency drift signals from the obtained fNIRS signals.

### Detection of Initial Dip

The initial dip will be detected through the vector phase analysis with dual threshold circles (Yoshino and Kato, 2012; Hong and Naseer, 2016; Zafar and Hong, 2018), see Figure 1. Vector phase analysis is a polar coordinate plane method defined by ΔHbO and ΔHbR as orthogonal vector components. Two other vector components, cerebral oxygen exchange (ΔCOE) and cerebral blood volume (ΔCBV), are obtained by rotating the vector coordinate system by 45° counterclockwise using the following equations (Yoshino et al., 2013; Khan et al., 2018).

(24) $$\begin{array}{l} \Delta \text{CBV}=\frac{1}{\sqrt{2}}\left(\Delta \text{HbO}+\Delta \text{HbR}\right), \end{array}$$

(25) $$\begin{array}{l} \Delta \text{COE}=\frac{1}{\sqrt{2}}\left(\Delta \text{HbR}-\Delta \text{HbO}\right). \end{array}$$

The magnitude and the phase of a vector *p* = (ΔHbO, ΔHbR) in the phase plane can be calculated as follows.

(26) $$\begin{array}{l} |p|=\sqrt{\Delta \text{Hb}{\text{O}}^{2}+\Delta \text{Hb}{\text{R}}^{2}}, \end{array}$$

(27) $$\begin{array}{l} ∠p=\tan^{-1}\left(\frac{\;\Delta \text{HbR}}{\;\Delta \text{HbO}}\right)=\tan^{-1}\left(\frac{\;\Delta \text{COE}}{\;\Delta \text{CBV}}\right)+45^{o}. \end{array}$$

The degree of oxygen exchange is defined by the ratio of ΔCOE and ΔCBV. Therefore, the oxygen exchange in the blood vessel is represented by the change in ΔCOE. Using the abovementioned four indices, eight phases are defined on the vector phase diagram, see Figure 1. Phases 1–5 (i.e., Phase 1: 0 < ΔHbR < ΔHbO, ΔCOE < 0 < ΔCBV; Phase 2: 0 < ΔHbO < ΔHbR, 0 < ΔCOE < ΔCBV; Phase 3: ΔHbO < 0 < ΔHbR, 0 < ΔCBV < ΔCOE; Phase 4: ΔHbO < 0 < ΔHbR, ΔCBV < 0 < ΔCOE; Phase 5: ΔHbO < ΔHbR < 0, ΔCBV < 0 < ΔCOE) are defined as the initial dip phases because they reflect an increase in either ΔHbR or ΔCOE, whereas Phases 6 to 8 (i.e., Phase 6: ΔHbR < ΔHbO < 0, ΔCBV < ΔCOE < 0; Phase 7: ΔHbR < 0 < ΔHbO, ΔCOE < ΔCBV < 0; Phase 8: ΔHbR < 0 < ΔHbO, ΔCOE < 0 < ΔCBV) are defined as HR phases. If there are no threshold circles in the vector diagram, the resting-state fluctuation and large fluctuations of ΔHbO and ΔHbR signals during the task period with ΔCOE > 0 can easily be interpreted as an initial dip. Threshold circles (i.e., dual threshold circles) incorporated in the vector phase analysis help in minimizing the detection of false dips (Hong and Naseer, 2016; Zafar and Hong, 2018). The radius of the first (inner) threshold circle in Figure 1 was determined during the resting state period as follows (Hong and Naseer, 2016).

(28) $$\begin{array}{l} r_{1}=\max\left(\sqrt{\Delta {\text{HbO}}_{resting}^{2}+\Delta {\text{HbR}}_{resting}^{2}}\right). \end{array}$$

The single (inner) threshold circle can help to separate the resting-state fluctuation from the initial dip and task-related HR. However, a large fluctuation of ΔHbO and ΔHbR above the threshold circle can still falsely be interpreted as an initial dip. Therefore, a second (outer) threshold circle as an upper bound is drawn on the vector diagram to separate large ΔHbO and ΔHbR fluctuations from the initial dip. The radius for the second threshold circle was determined using the following equation (Zafar and Hong, 2018).

(29) $$\begin{array}{l} r_{2}=r_{1}+0.3\left(p_{1}+\text{SD}\right), \end{array}$$

where *p*_1_ and SD are the peak value and standard deviation of the averaged ΔHbO trial over several trials from the most active channel, where the most active channel means the channel that shows the largest difference between the maximum ΔHbO values in the resting state and the HR of the first trial during the training stage. The initial dips are detected if the trajectory lies in any phase from Phase 3 to Phase 5 and remains within the two threshold circles within first 2–4 s of the task period, and it moves to either Phases 7 or 8, after 2–4 s. The first (i.e., inner) threshold circle is used to detect the time instance of the occurrence of an initial dip and the HR. Any trajectory going outside the secondary threshold circle is considered as a false dip or noise.

## Results

**Figure 4:**
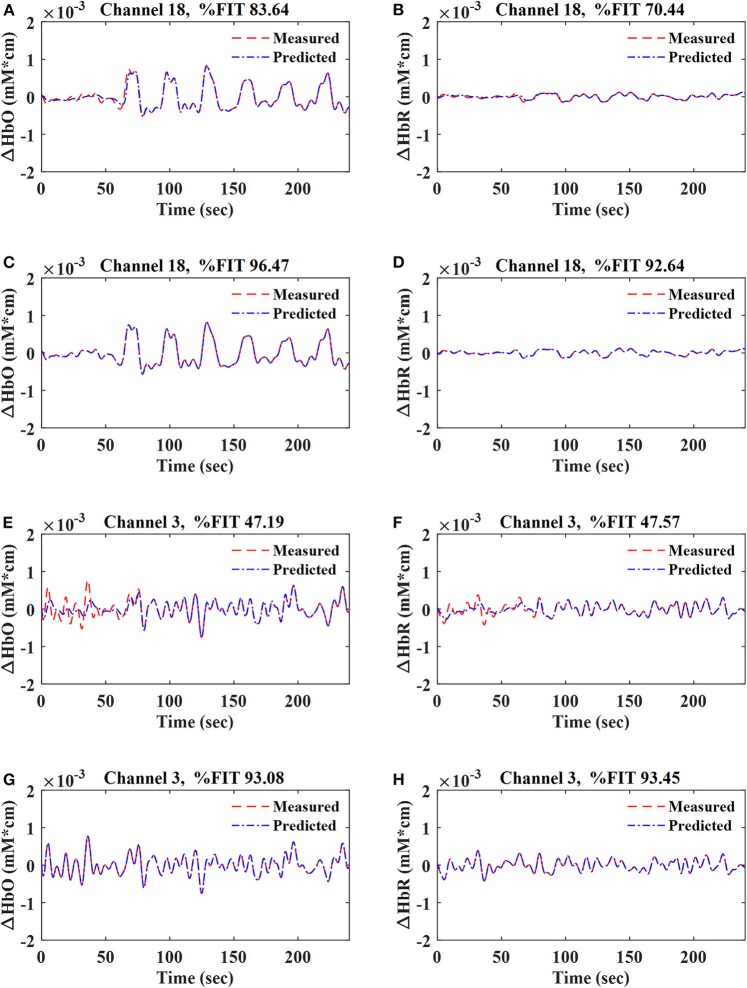
Fitting of ΔHbO and ΔHbR signals for active (Ch. 18) and non-active (Ch. 3) channels at *q* = 1 for Sub. 1: **(A,B,E,F)** were obtained by using the RLS method; **(C,D,G,H)** were obtained by using the Gaussian-kernel RLS method.

The data during the resting-state and the first session were used in the training stage, whereas the second session data were used to test the proposed method. The parameters of the Gaussian (i.e., σ), polynomial (i.e., *c* and *p*), and sigmoid (i.e., *s* and *t*) kernels were determined iteratively, and the value with the maximum %FIT for each kernel was selected for further analysis. Using the data of 792 channels [i.e., 11 subjects × (36 HbO + 36 HbR)], the values of parameters σ, *c, p, s*, and *t* for the Gaussian, polynomial, and sigmoid kernels were found to be 1 in the training stage. For the regularization parameter (*R*), several different values were tested through trial and error, and *R* = 10^−8^ was found to achieve the best fitting (i.e., %FIT) of the predicted signals. *R* > 10^−8^ did not affect the %FIT of the predicted signals, but lower values decreased %FIT. Figure 4 shows the fitting of the one-step-ahead predicted ΔHbO and ΔHbR signals on top of the measured signals (ΔHbO, ΔHbR) using the RLS method and the Gaussian-kernel RLS method for both active (i.e., Ch. 18) and non-active (i.e., Ch. 3) channels of Subject 1, respectively.

Tables 1–4 reports the %FIT of ΔHbO and ΔHbR for individual subjects using RLS and KRLS. The statistical significance of the %FIT was verified using two-sample *t*-tests. Signal information in the predicted signals (i.e., %FIT) significantly decreases (*p* < 0.05) as the step size increases. Table 5 shows a comparison of the averaged %FITs of RLS and KRLS with the Gaussian, polynomial, and sigmoid kernels for different *q-*step-ahead predicted fNIRS (ΔHbO, ΔHbR) signals.

**Table 1:** Averaged %FIT for ΔHbO and ΔHbR over all channels using RLS after training and testing.

**Sub**.	**%FIT**									
	***q*** **= 1 (0.1 s)**		***q*** **= 5 (0.54 s)**		***q*** **= 10 (1.08 s)**		***q*** **= 15 (1.63 s)**		***q*** **= 20 (2.17 s)**	
	**ΔHbO**	**ΔHbR**	**ΔHbO**	**ΔHbR**	**ΔHbO**	**ΔHbR**	**ΔHbO**	**ΔHbR**	**ΔHbO**	**ΔHbR**
1	69.8 ± 8.6	68.3 ± 6.3	66.6 ± 9.3	64.9 ± 6.9	62.6 ± 10.0	60.8 ± 7.5	59.2 ± 10.6	57.2 ± 8.1	56.6 ± 10.9	54.2 ± 8.5
2	65.5 ± 9.3	63.7 ± 7.4	61.6 ± 10.1	59.7 ± 8.1	57.1 ± 11.2	54.9 ± 8.9	52.9 ± 12.1	50.5 ± 9.6	49.6 ± 12.8	46.9 ± 10.2
3	67.0 ± 6.6	65.7 ± 5.4	63.2 ± 7.2	61.6 ± 5.9	58.7 ± 7.8	56.9 ± 6.5	54.8 ± 8.4	52.8 ± 7.1	51.5 ± 8.9	49.4 ± 7.5
4	69.4 ± 9.8	68.7 ± 6.5	66.0 ± 10.4	65.3 ± 7.0	61.9 ± 11.2	61.3 ± 7.5	58.2 ± 11.8	57.7 ± 8.4	55.0 ± 12.3	54.6 ± 8.4
5	65.4 ± 7.3	64.4 ± 6.2	61.6 ± 7.8	60.6 ± 6.6	57.1 ± 8.4	55.9 ± 7.2	53.1 ± 8.9	51.7 ± 7.8	49.7 ± 9.3	48.2 ± 8.2
6	73.0 ± 9.4	69.4 ± 7.5	70.1 ± 10.4	66.1 ± 8.3	66.5 ± 11.6	62.1 ± 9.2	63.3 ± 12.6	58.6 ± 10.1	60.5 ± 13.4	55.6 ± 10.8
7	76.1 ± 6.1	75.3 ± 4.3	73.2 ± 6.4	72.5 ± 4.7	69.8 ± 6.8	69.2 ± 5.2	66.7 ± 7.3	66.2 ± 5.6	64.1 ± 7.7	63.6 ± 6.0
8	73.6 ± 8.4	67.9 ± 6.1	70.5 ± 8.9	64.4 ± 6.6	66.9 ± 9.6	60.2 ± 7.4	63.7 ± 10.2	56.5 ± 8.1	61.1 ± 10.6	53.5 ± 8.6
9	68.8 ± 6.9	64.9 ± 4.5	65.4 ± 7.4	61.2 ± 4.8	61.5 ± 8.1	56.7 ± 5.2	58.0 ± 8.5	52.8 ± 5.4	55.1 ± 8.9	49.6 ± 5.7
10	76.1 ± 9.5	71.3 ± 7.1	73.2 ± 10.2	67.9 ± 7.7	69.7 ± 11.0	63.9 ± 8.5	66.4 ± 11.6	60.3 ± 9.3	63.5 ± 11.9	57.2 ± 9.9
11	70.1 ± 8.1	66.8 ± 6.0	67.0 ± 8.8	63.2 ± 6.5	63.2 ± 9.5	58.9 ± 7.1	59.9 ± 10.1	55.2 ± 7.6	57.2 ± 10.4	52.2 ± 8.1
Mean ± SD	70.4 ± 8.2	67.9 ± 6.1	67.1 ± 8.8	64.3 ± 6.6	63.2 ± 9.6	60.1 ± 7.3	59.7 ± 10.2	56.3 ± 7.9	56.7 ± 10.9	53.2 ± 8.4

**Table 2:** Averaged %FIT for ΔHbO and ΔHbR over all channels using KRLS with the Gaussian kernel after training and testing.

**Sub**.	**%FIT**									
	***q*** **= 1 (0.1 s)**		***q*** **= 5 (0.54 s)**		***q*** **= 10 (1.08 s)**		***q*** **= 15 (1.63 s)**		***q*** **= 20 (2.17 s)**	
	**ΔHbO**	**ΔHbR**	**ΔHbO**	**ΔHbR**	**ΔHbO**	**ΔHbR**	**ΔHbO**	**ΔHbR**	**ΔHbO**	**ΔHbR**
1	94.4 ± 0.8	93.6 ± 1.2	92.7 ± 1.6	91.5 ± 1.8	90.4 ± 2.7	89.0 ± 2.8	88.1 ± 3.7	86.6 ± 3.5	85.9 ± 4.6	84.4 ± 4.2
2	94.6 ± 1.0	92.2 ± 4.1	92.0 ± 2.2	89.4 ± 4.2	88.7 ± 4.1	85.5 ± 4.6	85.5 ± 5.9	81.9 ± 5.6	82.7 ± 7.5	78.8 ± 6.7
3	94.3 ± 0.7	91.2 ± 4.0	91.9 ± 1.7	88.4 ± 3.8	88.5 ± 3.1	84.9 ± 4.1	85.3 ± 4.5	81.7 ± 4.6	82.4 ± 5.7	78.9 ± 5.3
4	94.7 ± 1.0	92.8 ± 1.8	92.9 ± 1.3	90.9 ± 2.2	90.4 ± 2.7	88.3 ± 3.0	87.8 ± 4.1	85.9 ± 3.8	85.4 ± 5.4	83.6 ± 4.5
5	94.4 ± 2.1	92.9 ± 3.2	92.3 ± 2.1	90.5 ± 3.1	89.5 ± 2.6	86.9 ± 3.3	86.7 ± 3.6	83.6 ± 4.0	84.3 ± 4.6	80.6 ± 4.6
6	94.8 ± 0.9	93.1 ± 2.1	93.3 ± 1.6	91.1 ± 2.6	91.2 ± 2.6	88.5 ± 3.3	88.9 ± 3.8	85.9 ± 4.1	86.6 ± 5.0	83.6 ± 4.9
7	95.1 ± 0.9	94.9 ± 0.8	93.2 ± 1.4	93.1 ± 1.2	90.7 ± 2.5	90.8 ± 2.0	88.3 ± 4.0	88.6 ± 2.7	86.1 ± 4.6	86.5 ± 3.5
8	94.6 ± 0.9	92.2 ± 2.4	92.2 ± 1.6	89.9 ± 2.8	89.5 ± 2.5	87.1 ± 3.6	87.1 ± 3.4	84.0 ± 5.4	84.9 ± 4.2	82.1 ± 5.0
9	94.3 ± 0.6	93.9 ± 0.5	92.4 ± 1.4	92.0 ± 1.1	89.8 ± 2.4	89.1 ± 2.2	87.3 ± 3.3	86.3 ± 3.4	85.0 ± 4.2	83.6 ± 4.5
10	95.4 ± 1.0	94.4 ± 1.4	93.7 ± 1.4	91.8 ± 1.4	91.5 ± 2.4	88.7 ± 2.2	89.3 ± 3.4	85.8 ± 3.5	87.5 ± 4.3	83.3 ± 4.7
11	94.4 ± 1.1	93.6 ± 1.1	92.8 ± 1.6	91.7 ± 1.3	90.7 ± 2.4	89.1 ± 2.1	88.5 ± 3.4	86.5 ± 3.1	86.6 ± 4.2	84.1 ± 4.1
Mean ± SD	94.6 ± 1.0	93.2 ± 2.1	92.7 ± 1.6	90.9 ± 2.3	90.1 ± 2.7	88.0 ± 3.0	87.5 ± 3.9	85.2 ± 4.0	85.2 ± 4.9	82.7 ± 4.7

**Table 3:** Averaged %FIT for ΔHbO and ΔHbR over all channels using KRLS with the polynomial kernel after training and testing.

**Sub**.	**%FIT**									
	***q*** **= 1 (0.1 s)**		***q*** **= 5(0.54 s)**		***q*** **= 10 (1.08 s)**		***q*** **= 15 (1.63 s)**		***q*** **= 20 (2.17 s)**	
	**ΔHbO**	**ΔHbR**	**ΔHbO**	**ΔHbR**	**ΔHbO**	**ΔHbR**	**ΔHbO**	**ΔHbR**	**ΔHbO**	**ΔHbR**
1	93.5 ± 2.4	92.6 ± 2.4	91.6 ± 3.4	90.6 ± 2.6	89.1 ± 5.1	88.1 ± 3.2	86.5 ± 6.6	85.7 ± 3.8	84.1 ± 7.9	83.6 ± 4.3
2	94.3 ± 0.9	87.5 ± 14.1	91.7 ± 2.1	84.7 ± 13.7	88.4 ± 4.0	81.0 ± 13.4	85.2 ± 5.7	77.5 ± 13.4	82.4 ± 7.4	74.4 ± 13.7
3	94.0 ± 1.2	83.1 ± 19.7	91.4 ± 1.9	80.4 ± 19.7	88.1 ± 3.2	77.1 ± 20.0	84.8 ± 4.6	73.8 ± 20.4	81.9 ± 5.8	71.1 ± 21.0
4	94.5 ± 1.1	89.5 ± 7.3	92.6 ± 1.5	87.6 ± 7.3	89.9 ± 2.7	85.1 ± 7.4	87.3 ± 4.0	82.5 ± 7.7	84.8 ± 5.2	80.3 ± 8.1
5	94.2 ± 1.8	90.5 ± 8.0	91.9 ± 2.2	87.8 ± 7.6	89.1 ± 2.3	84.2 ± 7.3	85.7 ± 4.5	80.8 ± 7.2	83.1 ± 4.9	78.0 ± 7.3
6	94.7 ± 0.8	91.8 ± 4.9	93.3 ± 1.5	89.7 ± 5.2	91.2 ± 2.5	87.1 ± 5.8	89.0 ± 3.7	84.4 ± 6.6	86.7 ± 4.8	82.1 ± 7.5
7	95.1 ± 0.9	94.1 ± 2.9	93.1 ± 1.4	92.2 ± 2.8	90.6 ± 2.4	89.9 ± 3.1	88.2 ± 3.5	87.6 ± 3.4	86.0 ± 4.5	85.5 ± 3.9
8	94.5 ± 0.9	91.2 ± 4.3	92.1 ± 1.6	88.9 ± 4.5	89.3 ± 2.5	86.0 ± 4.9	86.9 ± 3.4	83.2 ± 5.5	84.8 ± 4.2	80.8 ± 6.1
9	94.1 ± 0.9	93.6 ± 0.8	92.2 ± 1.5	91.5 ± 1.3	89.5 ± 2.4	88.5 ± 2.9	86.9 ± 3.4	85.5 ± 3.6	84.6 ± 4.3	82.8 ± 4.7
10	95.3 ± 0.9	93.8 ± 2.4	93.6 ± 1.4	91.2 ± 2.2	91.4 ± 2.4	88.1 ± 2.8	89.4 ± 3.4	85.2 ± 3.8	87.4 ± 4.8	82.6 ± 4.9
11	94.4 ± 0.8	93.3 ± 1.6	92.8 ± 1.4	91.3 ± 1.8	90.6 ± 2.3	88.7 ± 2.5	88.4 ± 3.1	86.1 ± 3.4	86.4 ± 3.8	83.8 ± 4.3
Mean ± SD	94.4 ± 1.1	91.0 ± 6.2	92.4 ± 1.8	88.7 ± 6.2	89.7 ± 2.9	85.8 ± 6.7	87.1 ± 4.2	82.9 ± 7.2	84.7 ± 5.2	80.5 ± 7.8

**Table 4:** Averaged %FIT for ΔHbO and ΔHbR over all channels using KRLS with the sigmoid kernel after training and testing.

**Sub**.	**%FIT**									
	***q*** **= 1 (0.1 s)**		***q*** **= 5 (0.54 s)**		***q*** **= 10 (1.08 s)**		***q*** **= 15 (1.63 s)**		***q*** **= 20 (2.17 s)**	
	**ΔHbO**	**ΔHbR**	**ΔHbO**	**ΔHbR**	**ΔHbO**	**ΔHbR**	**ΔHbO**	**ΔHbR**	**ΔHbO**	**ΔHbR**
1	86.3 ± 13.2	66.1 ± 24.5	84.1 ± 12.9	64.2 ± 24.2	81.5 ± 12.7	61.8 ± 23.9	78.9 ± 12.6	59.5 ± 23.7	76.7 ± 12.6	57.4 ± 23.6
2	87.5 ± 9.7	63.6 ± 26.2	84.5 ± 9.2	60.9 ± 25.1	80.8 ± 9.1	57.4 ± 23.8	77.3 ± 9.2	54.1 ± 22.8	74.2 ± 9.7	51.2 ± 22.1
3	84.0 ± 13.2	53.8 ± 27.3	81.0 ± 12.8	51.5 ± 26.6	77.2 ± 12.4	48.7 ± 25.8	73.7 ± 12.3	45.8 ± 25.1	70.7 ± 12.3	43.4 ± 24.5
4	86.8 ± 13.6	61.6 ± 27.1	84.6 ± 13.5	59.7 ± 26.8	81.7 ± 13.4	57.2 ± 26.6	78.9 ± 13.3	54.7 ± 26.4	76.3 ± 13.4	52.5 ± 26.3
5	89.1 ± 6.2	70.2 ± 19.1	86.4 ± 6.1	67.8 ± 18.6	82.9 ± 6.2	64.6 ± 18.0	79.7 ± 6.6	61.7 ± 27.5	76.8 ± 7.3	59.1 ± 17.3
6	87.9 ± 10.7	73.9 ± 22.9	86.2 ± 10.5	72 ± 22.6	83.8 ± 10.4	69.4 ± 22.2	81.4 ± 10.4	66.9 ± 21.9	79.1 ± 10.5	64.7 ± 21.7
7	94.0 ± 3.2	84.7 ± 16.7	91.8 ± 3.4	82.8 ± 16.4	89.2 ± 3.8	80.4 ± 16.1	86.6 ± 4.4	78.1 ± 15.8	84.2 ± 5.1	76.0 ± 15.7
8	84.3 ± 16.5	66.8 ± 20.1	82.1 ± 16.1	64.8 ± 19.7	79.4 ± 15.6	62.2 ± 19.2	77.1 ± 15.3	59.7 ± 18.8	74.9 ± 15.0	57.4 ± 18.5
9	91.1 ± 5.8	82.3 ± 13.4	88.7 ± 5.5	79.8 ± 13.0	85.8 ± 5.2	76.6 ± 12.6	83.1 ± 5.2	73.7 ± 12.3	80.8 ± 5.4	71.2 ± 12.2
10	90.3 ± 6.4	75.4 ± 18.4	88.4 ± 6.3	73.1 ± 17.8	86.1 ± 6.3	70.2 ± 17.2	83.8 ± 6.5	67.5 ± 17.0	81.7 ± 7.0	65.1 ± 16.9
11	91.3 ± 4.5	76.4 ± 22.7	89.4 ± 4.5	74.0 ± 22.2	86.9 ± 4.6	70.9 ± 21.5	84.5 ± 4.9	68.1 ± 21.1	82.4 ± 5.2	65.5 ± 20.9
Mean ± SD	88.4 ± 9.3	70.4 ± 21.6	86.1 ± 9.2	68.2 ± 21.2	83.2 ± 9.1	65.4 ± 20.6	80.4 ± 9.2	62.7 ± 21.1	77.9 ± 9.4	60.3 ± 19.9

**Table 5:** Comparison of the averaged %Fits of RLS and KRLS (Gaussian, polynomial, sigmoid) for different *q*-step-ahead predicted ΔHbO and ΔHbR signals after training and testing.

***q*-step**	**%Fit of fNIRS signals**							
	**ΔHbO**				**ΔHbR**			
	**RLS**	**KRLS with Gaussian**	**KRLS with polynomial**	**KRLS with sigmoid**	**RLS**	**KRLS with Gaussian**	**KRLS with polynomial**	**KRLS with sigmoid**
1 (0.1 s)	70.4 ± 8.3	94.6 ± 1.0	94.4 ± 1.1	88.4 ± 9.3	67.9 ± 6.1	93.2 ± 2.1	91.0 ± 6.2	70.4 ± 21.6
5 (0.54 s)	67.1 ± 8.8	92.7 ± 1.6	92.4 ± 1.8	86.1 ± 9.2	64.3 ± 6.6	90.9 ± 2.3	88.7 ± 6.2	68.2 ± 21.2
10 (1.08 s)	63.2 ± 9.6	90.1 ± 2.7	89.7 ± 2.9	83.2 ± 9.1	60.1 ± 7.3	88.0 ± 3.0	85.8 ± 6.7	65.4 ± 20.6
15 (1.63 s)	59.7 ± 10.2	87.5 ± 3.9	87.1 ± 4.2	80.4 ± 9.2	56.3 ± 7.9	85.2 ± 4.0	82.9 ± 7.2	62.7 ± 21.1
20 (2.17 s)	56.7 ± 10.9	85.2 ± 4.9	84.7 ± 5.2	77.9 ± 9.4	53.2 ± 8.4	82.7 ± 4.7	80.5 ± 7.8	60.3 ± 19.9

**Figure 5:**
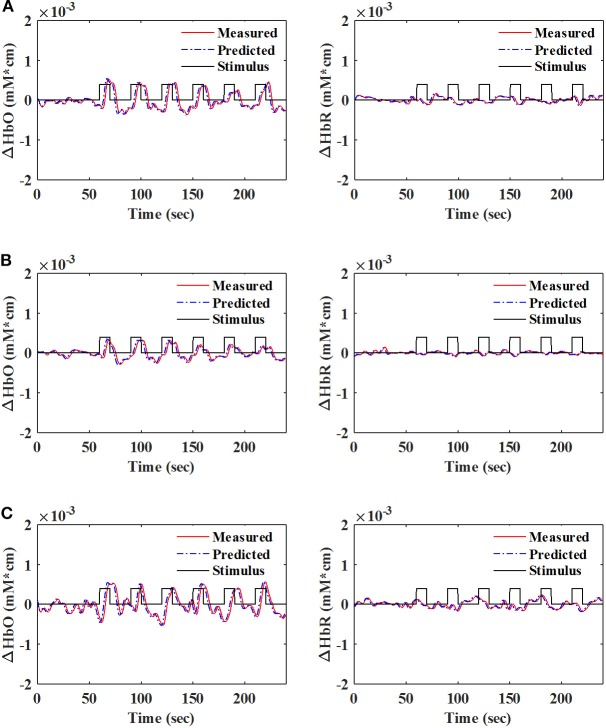
Measured and 1.63 s ahead predicted signals (*q* = 15) of HbO (left) and HbR (right) with the Gaussian-kernel RLS algorithm: **(A)** Sub. 1 (Ch. 21), **(B)** Sub. 8 (Ch. 18), and **(C)** Sub. 10 (Ch. 30).

A number of previous studies reported that the peak of the initial dip occurred at approximately 1.9–2.5 s (Hu and Yacoub, 2012; Zafar and Hong, 2017). Therefore, *q* = 15 (i.e., 1.63 s since the sampling frequency was 9.19 Hz in this study) was selected for further analysis. Table 1 shows that KRLS with the Gaussian kernel yielded the best fitting (*p* < 0.05) for the 1.63 s ahead predicted ΔHbO (i.e., 87.5%) and ΔHbR (i.e., 85.2%) signals as compared to all other methods. Therefore, the Gaussian-kernel RLS was further used for reducing the delay in detecting initial dips in the fNIRS signals. Figure 5 shows the 15-step-ahead predicted ΔHbO and ΔHbR signals of active channels for different subjects. It can be clearly seen that the predicted signals are well-ahead (i.e., blue dotted lines) and perfectly tracking the measured signals (solid red line).

**Figure 6:**
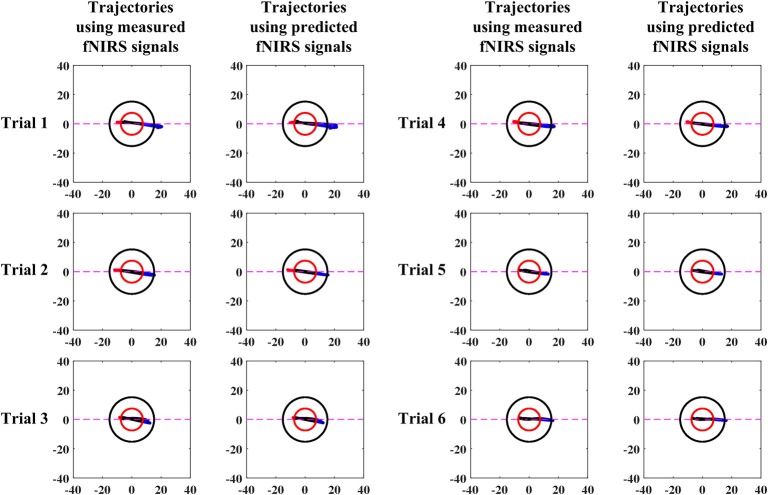
Comparison between measured and predicted (1.63 s) signals (Sub. 3, Ch. 21).

A comparison of vector-phase trajectories using measured and 1.63 s ahead predicted fNIRS signals for Subject 3 is shown in Figure 6. Table 6 shows the times of initial dip detection using 15-step-ahead predicted ΔHbO and ΔHbR signals for active channels of all subjects.

**Table 6:** Time of initial dip detection using 1.63 s (*q* = 15) ahead prediction.

**Sub**.	**Channel**	**Time of initial dip detection (sec)**					
		**Trial 1**	**Trial 2**	**Trial 3**	**Trial 4**	**Trial 5**	**Trial 6**
1	21	0.65	0.11	0.11	0.11	Not detected	Not detected
2	18	0.11	0.11	0.21	0.65	Not detected	0.65
3	21	0.11	0.11	Not detected	0.11	Not detected	0.11
4	29	0.11	0.11	0.11	0.32	0.11	0.21
5	33	0.11	0.32	0.21	Not detected	0.21	0.11
6	29	0.11	0.43	Not detected	Not detected	Not detected	0.11
7	17	0.11	Not detected	0.21	0.21	0.11	Not detected
8	21	Not detected	0.11	Not detected	0.32	0.11	Not detected
9	17	Not detected	0.21	0.43	0.21	0.21	Not detected
10	29	0.11	0.32	0.65	0.65	0.11	0.11
11	33	0.11	Not detected	0.11	0.11	Not detected	0.11

## Discussions

The newly emerging neuroimaging modality (i.e., fNIRS) has a disadvantage of inherent onset delay from neuronal activation, which limits its application for rapid BCIs. To overcome this, the use of a kernel method for *q*-step-ahead prediction of fNIRS signals was proposed for the first time. The novelty of this study lies in using an online prediction scheme to reduce this onset delay for online applications.

A previous study by Hong and Naseer (2016) could reduce the delay in detecting initial dip in fNIRS signals to approximately 0.9 s using an offline *q*-step-ahead ARMAX model-based prediction scheme. Our results (Tables 1–5) reveal that the fitting accuracy of the *q*-step-ahead predicted signals decreased significantly (*p* < 0.05) with the increase of prediction step sizes. Therefore, the selection of a proper step size is very crucial to ensure that the predicted signals contain the maximum information of the measured signals.

In this study, a linear combination of three gamma functions was used (i.e., dHRF, see Figure 2). Most early studies used only s two-gamma-function dHRF to analyze the fNIRS time-series (Abdelnour and Huppert, 2009; Ye et al., 2009; Hu et al., 2010). A key drawback in using two gamma functions is that the initial dip duration is neglected in the estimation/prediction process. This limitation was overcome by using three gamma functions, which provides an extra degree of freedom by including the initial dip in the dHRF model for better estimation/prediction of the fNIRS signal.

The KRLS algorithm improves the fitting of the predicted signals as compared to the RLS algorithm by moving from the input space to the transformed feature space, i.e., a high dimensional space (see Tables 1–5). The non-linear relationship in the data cannot be adequately modeled by using linear regression techniques. The advantage of moving to a higher dimensional space is that there is a high probability that the data corresponds to a linear model, and it can be solved using the linear algorithms (Liu et al., 2010). Regarding the kernels, the Gaussian kernel yielded the best fitting of the predicted ΔHbO (i.e., 87.5%) and ΔHbR (i.e., 85.2%) signals at *q* = 15 step-ahead. The polynomial kernel also yielded good results for the ΔHbO signals, but the fitting slightly decreased for the ΔHbR signals. In contrast, the fitting of both ΔHbO and ΔHbR signals significantly decreased for the sigmoid kernel. Furthermore, the fitting of the predicted ΔHbR signals was lower than that of the predicted ΔHbO.

Early studies reported that the peak of initial dip occurred around 1.9–2.5 s (Malonek and Grinvald, 1996; Yacoub and Hu, 2001; Yacoub et al., 2001; Hu and Yacoub, 2012; Zafar and Hong, 2017). From this viewpoint, 1.63 s ahead prediction was selected in this study for an early detection of initial dips. Nevertheless, the peak of an initial dip depends on various factors, such as the type of task performed, the duration of the task period, and the brain area under investigation. The trajectories (Figure 6) for both measured and predicted signals were almost the same, showing that the predicted signals were well-tracking the measured signals. However, if the fitting of the predicted signal is not adequate, the trajectory can lead to a wrong decision regarding the detection of initial dip or HR. With 1.63 s ahead prediction (Table 6), the initial dips were detected in minimum 0.11 s (maximum 0.65 s), which is much lower than that of Hong and Naseer (2016) (i.e., 0.9 s). Furthermore, the initial dip phenomenon did not occur in some trials. In the literature, this issue has been discussed considering several issues. One interesting report is that it is due to the use of caffeine before the experiment (Behzadi and Liu, 2006; Hong and Zafar, 2018). In addition, the detection time of the initial dip varies among trials and subjects (Hu et al., 2013).

Finally, this study demonstrated a step moving toward the development of a real-time BCI and a brain monitoring system using fNIRS. The significance of this study lies in the fast detection of activity-related responses in fNIRS signals. Even if an initial dip is not present, the inherent onset delay in the conventional HR can be reduced using the proposed *q*-step-head prediction scheme. Moreover, the use of *q*-step-head prediction with improved fitting can help in the hybridization of fNIRS with other rapid modalities such as EEG. Nevertheless, further research is still required to improve the fitting of the predicted fNIRS signals with an accuracy more than 90% using advanced signal processing (Ghafoor et al., 2017; Chen et al., 2018; Hong et al., 2018a) and adaptive algorithms (Iqbal et al., 2018; Nguyen Q. C. et al., 2018). In the future, other types of kernels should also be investigated for further improvement of the predicted fNIRS signals. The limitations of this study are as follows: (i) the order of the system (*a*_n_) and the input (*b*_n_) was set as 1 to ensure low computational complexity. Therefore, the optimal order of the system and the input for the prediction of fNIRS signals should be investigated further. (ii) Exogenous signals were excluded from the estimation/prediction process. These signals should be considered for further improvement of the predicted fNIRS signals in the future.

## Conclusion

In this study, the *q*-step-ahead prediction scheme based on KRLS was used to reduce the onset delay from the neuronal activation in fNIRS signals. fNIRS signals of right-hand finger tapping task acquired from the left motor cortex were used to evaluate the performance of the prediction scheme. The results show that the Gaussian kernel yields the best fitting for both ΔHbO (i.e., 87.5%) and ΔHbR (i.e., 85.2%) signals at *q* = 15 step ahead prediction (i.e., 1.63 s with the sampling frequency of 9.19 Hz). The application of the scheme was found to reduce the delay in detecting the initial dip. The improvement in the fitting of 1.63 s ahead predicted fNIRS signals enabled the detection of initial dip in 0.1 s. The reduction in the onset delay is a significant improvement in the development of real-time BCI applications using fNIRS.

## Data Availability Statement

The datasets analyzed in this article are not publicly available. Requests to access the datasets should be directed to kshong@pusan.ac.kr.

## Ethics Statement

The studies involving human participants were reviewed and approved by Pusan National University Institutional Review Board. The patients/participants provided their written informed consent to participate in this study.

## Author Contributions

AZ carried out the data processing and wrote the first draft of the manuscript. K-SH suggested the theoretical aspects of the current study, corrected the manuscript, and supervised the entire process from the beginning.

### Conflict of Interest

The authors declare that the research was conducted in the absence of any commercial or financial relationships that could be construed as a potential conflict of interest.
